# Cellular Signalling and Photobiomodulation in Chronic Wound Repair

**DOI:** 10.3390/ijms222011223

**Published:** 2021-10-18

**Authors:** Thobekile S. Leyane, Sandy W. Jere, Nicolette N. Houreld

**Affiliations:** Laser Research Centre, Faculty of Health Sciences, University of Johannesburg, P.O. Box 17011, Doornfontein 2028, South Africa; sadie.leyane@gmail.com (T.S.L.); sandywjere@gmail.com (S.W.J.)

**Keywords:** photobiomodulation, cellular signalling pathway, wound healing, calcium, MAPKs, JAK/STAT

## Abstract

Photobiomodulation (PBM) imparts therapeutically significant benefits in the healing of chronic wounds. Chronic wounds develop when the stages of wound healing fail to progress in a timely and orderly frame, and without an established functional and structural outcome. Therapeutic benefits associated with PBM include augmenting tissue regeneration and repair, mitigating inflammation, relieving pain, and reducing oxidative stress. PBM stimulates the mitochondria, resulting in an increase in adenosine triphosphate (ATP) production and the downstream release of growth factors. The binding of growth factors to cell surface receptors induces signalling pathways that transmit signals to the nucleus for the transcription of genes for increased cellular proliferation, viability, and migration in numerous cell types, including stem cells and fibroblasts. Over the past few years, significant advances have been made in understanding how PBM regulates numerous signalling pathways implicated in chronic wound repair. This review highlights the significant role of PBM in the activation of several cell signalling pathways involved in wound healing.

## 1. Introduction

Wound healing is a biological process that involves the replacement and/or restoration of devitalised and damaged tissue and is aimed at maintaining functional and anatomical continuity of the tissue [1]. However, the process is prone to interferences in the orderly sequence and expected time frame, resulting in the formation of non-healing chronic wounds [2].

A wound can be defined as any injury or a break/laceration in the skin resulting in the disruption of its functional and structural continuity and loss of epithelial integrity [3]. Based on the nature of the wound healing process, wounds can be classified into two categories, namely acute and chronic. Acute wounds progress through a predictable and orderly reparative process, including the healing rate and time for wound closure, and there is restoration of tissue integrity [4]. Chronic wounds are defined as an injury that fails to progress through the normal phases of healing. These wounds become detained in one or more of the wound repair phases, typically the inflammatory phase, without the institution of a continued functional and structural outcome [2]. Chronic wounds pose a major financial challenge on global healthcare systems, with costs reaching up to 25 billion USD annually in the United States [5,6].

Photobiomodulation (PBM), previously known as low-level laser therapy (LLLT), is defined as the application of non-ionising radiation in the form of light with the intent to heal. PBM employs the use of photons at a non-thermal irradiance to elicit photochemical and photophysical events to alter biological activity [7,8]. These non-ionising light sources can either be coherent light (lasers) or non-coherent light (broadband light sources and light-emitting diodes- LEDs), typically in the visible red or near-infrared (NIR) spectrum [7,8]. PBM is devoid of thermal and ablative mechanisms and exploits the absorption of light to affect a chemical change [9]. The unique properties of PBM have allowed for its use in the treatment of a variety of medical disorders, including the promotion of tissue repair and restoration and stimulation of chronic wound healing [10].

The dynamic nature of wound healing is orchestrated in four fundamental overlapping and interrelated cascades, namely haemostasis, inflammation, cellular proliferation, and tissue remodelling [11,12]. Wound healing follows instantly after an injury to the skin and proceeds by a systematised interaction of cellular and biochemical events executed by multiple cell types and tissues, and is tightly regulated by various chemokines, cytokines, and growth factors [3,13]. These growth factors, cytokines, and low molecular weight serum proteins regulate cellular responses and stimulate intracellular signalling pathways involved in the regulation of cell proliferation, differentiation, migration, and protein synthesis [4]. Intracellular signalling dynamics encode injury stimuli to coordinate tissue restoration reactions essential for effective wound healing [14]. This review article looks at the role PBM plays in the activation of several cell signalling pathways involved in wound healing. An overview of these effects can be seen in Table 1.

## 2. Phases of Wound Healing

### 2.1. Haemostatic Phase

This is the first phase in wound healing and is characterised by a vascular response [34]. It involves the constriction of blood vessels immediately after injury to restrict blood flow to the injured tissue [35]. There is aggregation of thrombocytes and platelets and migration of immune cells to the damaged tissue by chemotaxis in response to released cytokines and growth factors [36]. Apart from preventing excessive blood loss, the blood clot serves as a provisional protective barrier against invasion by microorganisms at the wound site [4]. Additionally, the clot creates a temporary environment suitable for cellular communication, proliferation, and migration for a timely wound restoration process, thus preserving the skin’s functional integrity [1]. This phase is typically completed within hours of the injury [37].

### 2.2. Inflammatory Phase

The second stage in wound healing, inflammation, is characterised by a cellular response, which is highlighted by the influx of white blood cells, mainly neutrophils and macrophages [3,35]. It is initiated when there is inhibition of local lymphatic function due to the extravasation of fluid to extracellular spaces as a result of the dilation of injured blood vessels [34]. This causes the cardinal signs associated with inflammation, namely heat, redness, swelling, and pain, at the location of the lesion. Additionally, neutrophils are responsible for the release of active antimicrobial constituents, such as proteases, that facilitate the clean-up of cell debris, bacteria, and foreign bodies, and the release of extracellular reactive oxygen species (ROS) at the injury site [35,38]. It is initiated within the first 24 h of injury. At the end of the inflammatory phase, inflammatory cells undergo apoptosis [3].

### 2.3. Proliferative Phase

The third stage in wound healing, the proliferative phase, features three specific stages, namely re-epithelialisation, neovascularisation, and granulation [4,34]. It is characterised by angiogenesis and the formation of collagen and extracellular matrix (ECM) complexes critical for proper repair [1]. Keratinocytes and fibroblasts are key cells involved in this phase and are responsible for the closure of the injured tissue [35]. Contraction and formation of fibrous tissue reduces the surface area of the wound, creating a viable epithelial barrier to activate keratinocytes and strengthen the wound [35]. Fibroblasts differentiate into specialised myofibroblasts that play a critical role in wound contraction and the synthesis and construction of ECM [38,39]. The fibrin matrix formed during the haemostatic phase is replaced with granulation tissue, which is rich in ECM compounds, hyaluronic acid, and fibronectin [40]. The proliferative phase occurs about four days after injury.

### 2.4. Remodelling Phase

The final phase, also termed the maturation phase, is characterised by the remodelling of collagen type III to collagen type I to attain maximum tensile strength [41]. Fibroblasts reorganise, degrade, and resynthesise the ECM to accomplish wound contraction [35]. Collagen and matrix macromolecules synthesised by fibroblasts are critical for supporting the structural maintenance of granulation tissue [38]. An attempt is made to regain normal tissue anatomy through the formation of avascular and acellular scar tissue [35]. Apoptosis of fibroblasts, and the disappearance of inflammatory cells and blood vessels, contributes to the formation of acellular and avascular scar tissue [42]. The cross-linking of collagen fibres reduces the thickness of the scar and increases its tensile strength [4]. This phase starts 21 days after injury and continues for up to two years or more.

## 3. Intracellular Signalling Stimulated by Injury

The wound restorative process is influenced through the action of intracellular kinases, transcription factors, chemokines, cytokines, growth factors, inhibitors, and their receptors [43]. There are several signalling pathways that are activated following tissue injury, including mitogen-activated protein kinases (MAPKs) and Janus kinase/signal transducer and activator of transcription (JAK/STAT). Deng et al. [29], Handly and Wollman [44], and Handly et al. [45] identified calcium (Ca^2+^) and extracellular signal-regulated kinase (ERK) signalling as the fastest transduction pathways to initiate wound healing. 

### 3.1. Activation of Calcium in Tissue Injury

Due to delayed activation of transcriptional machinery following injury, the first response depends on transcription-independent signalling pathways that can be stimulated quickly. These pathways involve Ca^2+^, ROS, and adenosine molecules [46]. In tissue injury, the elevation of intracellular Ca^2+^ has been associated with the modulation of keratinocyte and fibroblast cell activities including proliferation, migration, differentiation, and gene expression [14,47]. Ca^2+^ plays a key role in skin homeostasis, and in injured tissue, increased Ca^2+^ in the granular layer of the epidermis has been shown to increase cell proliferation and migration [48]. The metabolism of tissue Ca^2+^ is determined by membrane-bound and intracellular Ca^2+^ binding proteins, including calmodulin and cadherins [49]. A study conducted by Yoo et al. [50] found that wounding the epithelia of zebrafish larval tail fin rapidly activated the Src family kinase (SFK) and Ca^2+^ signalling. Additionally, Klepeis et al. [51], Shabir and Southgate, [52], and Chifflet et al. [53] found elevated intracellular Ca^2+^ in cells bordering the injured area. 

Immediately following injury, there is activation of intracellular Ca^2+^ orchestrated by the release of inositol-1,4,5-trisphosphate (IP3), releasing the IP3-sensitive Ca^2+^ stores from the endoplasmic reticulum (ER) into the site of injury [14,54]. There is phosphorylation of P2Y purinergic G-coupled protein receptors by ATP released from wounded cells, leading to the activation of phospholipase C (PLC), which results in elevated IP3 levels [45,55]. IP3 binds to IP3 receptors (IP3R) on the ER, maintaining intracellular Ca^2+^ stores in a depleted state [56]. The depletion of Ca^2+^ stores occurs due of their release into the cytoplasm, and the initial Ca^2+^ response from healthy neighbouring cells is activated by ATP released from injured cells [44]. However, the Ca^2+^ response may be propagated further through the GAP junction from the injury site [44]. Additionally, elevation of cytosolic Ca^2+^ occurs when ATP, initially released from injured cells, interacts with extracellular receptors found on the surfaces of surrounding healthy cells [44]. Elevated cellular Ca^2+^ affects numerous signalling pathways, including the MAPK pathway [57]. 

### 3.2. Activation of Mitogen-Activated Protein Kinases (MAPKs) in Tissue Injury

MAPKs are a family of serine/threonine kinases located in the cytoplasm of mammalian cells and are involved in the regulation of a diverse range of physiological processes following activation through a broad array of extracellular stimuli [31,57,58]. Viruses, inflammatory cytokines, growth factors, and physical injury can activate the MAPK signalling pathway, transmitting signals from the cell membrane to the nucleus and resulting in the activation of physiological processes including the induction of apoptosis, cell differentiation and proliferation, oxidative stress, and inflammation [58,59,60]. MAPKs play an essential role in regulating osteoblast differentiation, ECM deposition, and mineralisation in response to osteoblastic ligands including Wnt proteins, transforming growth factor-beta (TGF-β), parathyroid hormone (PTH), and bone morphogenetic protein 2 (BMP2) [31,61]. The MAPK family consists of four subfamilies, namely, ERK 1/2, p38 MAPK, c-Jun-terminal kinase (JNK 1/2), and ERK 5 [30,57]. However, only ERK1/2, JNK1/2, and p38 MAPK have been widely studied. 

**Figure 1:**
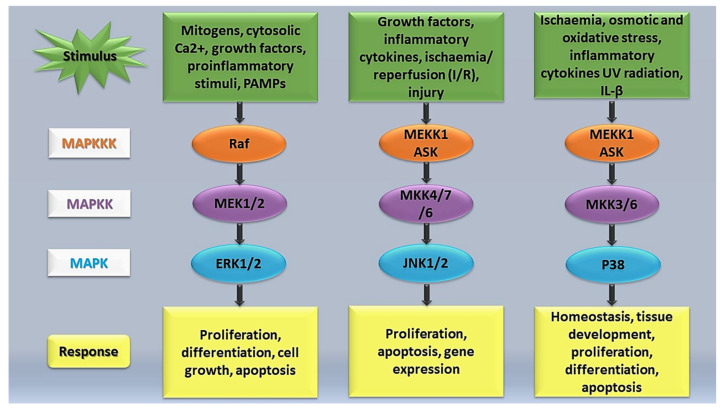
Simplified overview of the MAPK signalling pathway.

MAPK signalling pathways (Figure 1) have been implicated in the regulation of cellular stresses, such as inflammation, and several human diseases including cancer and cerebrovascular and cardiovascular diseases [58]. During tissue injury, MAPK signalling plays a vital role in transducing extracellular signals to phenotypic cellular responses. According to Petersen et al. [62], the MAPK signalling pathway undergoes phosphorylation by upstream kinases in response to injury. MAPK subfamilies have been shown to be major players in regulating the inflammatory phase of the wound restoration process [63]. A study conducted by Arthur and Ley [64] found that JKN, ERK, and p38 were activated following an inflammatory stimulation, leading to the transcriptional activation of selected genes that work together to mediate the inflammatory response. The various MAPKs have diverse roles and therefore generate different biological effects. Arthur and Ley [64] highlighted that some MAPKs, such as p38α, ERK1/2, and JNK1/2, play a critical role in innate immunity reactions. Activation of these MAPKs comprises a three-tiered enzymatic cascade, namely, MAPK, MEK/MAPK kinase (MAPKK), a MAPK activator, and MEKK/MAPKK kinase (MAPKKK), a MAPKK activator [65,66]. Activation of MAPKKK is achieved through upstream signalling proteins, and after its activation, MAPKK is phosphorylated and activated [57,65]. Activated MAPKK phosphorylates and ultimately activates the MAPK signalling network, instigating a series of physiological and pathological responses [57].

#### 3.2.1. ERK1/2 MAPK Pathway

The ERK1/2 MAPK pathway is one of the main regulators of cell growth and cycle. ERK1 and ERK2 are located in the cytoplasm and are often activated by extracellular mitogens [66]. ERK1/2 signalling is one of the most studied pathways that participates in regulating apoptosis, cell growth, proliferation, and differentiation [67]. Previous studies found that the activation of the ERK pathway regulates cellular migration in tissue repair [57,65]. Additionally, extracellular stimuli, such as oxidative stress, an elevation in cytosolic Ca^2+^ levels, pro-inflammatory stimuli, pathogen-associated molecular patterns (PAMPs), and growth factors, such as platelet-derived growth factor (PDGF) and heparin-binding EGF-like growth factor (HB-EGF), induces phosphorylation of the ERK1 and ERK2 pathway [57,68,69]. Growth factors can be released through paracrine signalling systems in response to extracellular stimuli including damage signals [14]. The extracellular stimuli interact with and activates receptor tyrosine kinases (RTKs) and Ras [14,66]. Ras is known as an upstream signalling protein (a small GTP-binding protein), which plays a role in regulating ERK, JNK, and p-38 MAPK activities [65]. Activated Ras binds to Raf, which is then recruited to the plasma membrane from the cytoplasm, and its activation sets off the three-tiered enzymatic cascade (Raf-MEK-ERK signalling cascade) [14,70]. Phosphorylation of the ERK1/2 pathway is induced by MEK 1 (MAPKK1) and MEK2 (MAPKK2), which takes place at Thr185 and Tyr187 [57]. Activation of the ERK1/2 pathway ultimately results in the regulation of cell metabolism, function, and tissue regeneration [66].

#### 3.2.2. JNK MAPK Pathway

The JNKs, also known as stress-activated protein kinases, are responsible for regulating apoptosis and cell proliferation [57]. Unlike the ERK pathway, the JNK pathway experiences poor activation by mitogens. Activation of this pathway occurs in response to the dual phosphorylation on tyrosine or threonine residues in the activation loop (Thr183/Tyr185) [57,65]. Previous studies have elucidated the activation of the JNK pathway by growth factors, inflammatory cytokines, glomerulonephritides, as well as ischaemia/reperfusion (I/R) and hyperglycaemia [65,71]. The JNK pathway is phosphorylated by SEK1/MKK4/JNKK1, and MAPKK, and the activation of MKK4 is induced by MEKK1 [65]. Deng et al. [29] clarified that the phosphorylation of the JNK pathway is dependent on the expression of MEKK1. Thus, MEKK1-mediated activation of the JNK pathway plays an important role in wound repair and the migration of epithelial cells [29]. In vitro keratinocyte wound healing was observed through specific activation of JNK by MKK7 and MKK6 [30]. Both Ramet et al. [72] and Bosch et al. [73] used Drosophila to demonstrate the involvement of the JNK pathway in wound healing.

#### 3.2.3. P38 MAPK Pathway

p38 MAPK, like JNK, is referred to as a stress-activated protein kinase. In tissue injury, p38 MAPK regulates tissue homeostasis and development, inflammation, apoptosis, cell proliferation, differentiation, cytokine production, neuropathic pain, and cell survival [31,32,33]. p38 MAPK is widely distributed throughout the body and is activated by cellular stress from stimuli including heat shock, osmotic and oxidative stress, ischemia, interleukin-1 beta (IL-1β), and ultraviolet (UV) radiation [57,74]. Activation of p38 MAPK occurs in response to the dual phosphorylation on tyrosine or threonine residues and activated p38 MAPK translocates into the nucleus from the cytosol where they transcribe their respective target proteins [57,62]. Additionally, phosphorylation of p38 MAPK is achieved through upstream MAPKs, namely MAPKK3 (MKK3) and MAPKK6 (MKK6) [57]. Unlike the p38 MAPK pathway, ERK and JNK pathways are independent of MKK3 and MKK6. IL-6, IL-8, and tumour necrosis factor-alpha (TNFα) have been identified to be upregulated by p38 MAPK [65].

### 3.3. The Janus Kinase/Signal Transducer and Activator of the Transcription (JAK/STAT) Signalling Pathway

The JAK/STAT signalling pathway is the main transduction pathway responsible for coordinating a wide array of cellular responses of cytokines and growth factors [75]. These responses lead to critical biological processes, such as cellular proliferation, differentiation, survival, migration, apoptosis, and inflammation [75,76].

The JAK/STAT signalling pathway is stimulated by a variety of ligands and their receptors [76]. Activation of the JAK/STAT signalling pathway occurs when the ligand binds to their receptor subunits, causing multi-merisation to occur [77]. JAKs, which are tyrosine kinases, form a stable association with the cytoplasmic regions of type I and type II cytokine receptors, causing signal transduction through either homodimers, heterodimers, or heteromultimers [76,78]. Four JAKs exist in mammals, namely JAK1, JAK2, JAK3, and Tyk2 [79]. After multi-merisation, JAKs transphosphorylation is induced, leading to subsequent recruitment of latent cytoplasmic STATs to be phosphorylated, including receptor subunits and the STATs’ major substrates [78,80]. The seven members of the STAT family, namely STAT1, STAT2, STAT3, STAT4, STAT5A, STAT5B, and STAT6, bear highly conserved tyrosine residues in the C-terminus region, also referred to as the transactivation domain (TAD), that are phosphorylated by JAKs [78]. This authorises the homo- or hetero-dimerization of STATs through the conserved SH2 domain (Src homology 2) [78,79]. The dimerised STATs are then translocated to the nucleus by the importin through an α-5-dependent mechanism through the Ran nuclear import pathway [77]. The dimerised STATs bind to specific promoter sequences, repressing or inducing transcription of the target genes involved in cellular proliferation, differentiation, and apoptosis [78]. The recruitment of different JAKs and STATs is largely influenced by the signal, tissue, and cellular context engaged in the signalling episode [75]. 

Studies have described several negative regulators that modulate the function of the JAK/STAT signalling pathway, namely the suppressors of cytokine signalling (SOCS), protein inhibitors of activated STATs (PIAS), and protein tyrosine phosphatase (PTPs) [78,79]. Proteins of the PIAS family inhibit binding of activated STATs to specific DNA sequences by binding to the dimers of the activated STATs [78]. The PIAS proteins are also responsible for facilitating post-translational modifications of STATs [79]. Proteins of the SOCS family are competitors of STAT receptor binding [79]. SOCS proteins also recruit E3 ubiquitin ligases, causing proteasomal degradation [78]. Most important is the negative feedback loop of SOCS proteins, which is regulated by STATS, thus inhibiting JAK protein function [76,79]. Eight members of the SOCS family have been identified, namely SOCS1, SOCS2, SOCS3, SOCS4, SOCS5, SOCS6, SOCS7, and cytokine-inducible SH2 domain protein (CISH) [78]. Proteins of the SOCS family play a critical role in regulating growth factors and cytokines involved in the wound repair process [22]. A study conducted by Feng et al. [22] reported on the gene expression pattern of seven SOCS family members in healing/healed chronic wounds and non-healing chronic wounds. The results demonstrated significantly higher SOCS3 and 4 in non-healing chronic wounds as compared to healing/healed chronic wounds. In non-healing chronic wounds, there is upregulation of SOCS3 [22]. Linke and colleagues [81] found that the presence of SOCS3 disrupted keratinocyte proliferation and migration in epithelial repair of cutaneous wounds, contributing to the diminished wound healing process.

## 4. Photobiomodulation (PBM)

The earliest application of PBM was by Professor Endre Mester, a Hungarian physician, in 1967. He reported on how laser light exposure accelerated hair growth in shaven mice while studying the effects of irradiation on cancerous cell growth [82]. However, in the 1980s, Professor Karu proposed the use of laser light as a therapy to treat cellular failure [83], and since then, the application of light as a therapy has evolved and expanded drastically.

PBM uses low-intensity light over the injury site to reduce pain and inflammation and activate tissue repair [8]. It is a low-risk, non-invasive, pain-free, and cost-effective medical therapy for the effective management of conditions, such as non-healing chronic wounds [34,84]. There are well-entrenched reports on the range of clinical benefits induced by PBM, from the treatments on non-healing chronic wounds, ulcers, burn wounds, chronic pain, and immune modulation, to dermatological conditions, scarring, and athletic performance enhancements [7,85,86,87]. A study conducted by Rathnakar and colleagues [88] reported on the effectiveness of PBM at various wavelengths (632.8, 785, and 830 nm) on healing burn wounds in Swiss albino mice. The findings suggested that PBM at 830 nm (3 J/cm^2^) had a profound effect in regulating proliferation, neovascularisation, and re-epithelisation at a faster rate, leading to wound contraction as compared to wavelengths at 632 and 785 nm and untreated controls. This resulted in the healing of burn wounds. PBM results in faster wound contraction, increases re-epithelisation, and reforms connective tissue and collagen fibres in vitro, in vivo, and in animal studies [84]. Additionally, Beckmann et al. [84] states that PBM reduces the inflammatory reaction and causes an increase in the proliferation of fibroblast cells. Almeida-Lopes et al. [89] irradiated human gingival fibroblasts at different wavelengths (670, 780, 692, and 786 nm) at 2 J/cm^2^. The cells were evaluated two, four, and six days following irradiation. PBM was reported to improve fibroblast proliferation in vitro. Thus, it was concluded that a shorter radiation exposure results in higher proliferation of human gingival fibroblasts.

Despite several in vitro studies having been conducted successfully, there are many conflictions between clinical protocols with respect to wavelength, fluence, and delivery regimens that avert a stringent concord [34,85]. Another limiting factor of PBM is the lack of randomised and controlled clinical trials in wound healing to better understand the mechanism of PBM since there may be a variance between in vitro and in vivo environments [84]. Studies have documented the molecular and cellular alterations in tissues exposed to PBM, though the mechanism of action is not yet fully understood. Therefore, obstacles, such as the shortage of translational research with consistent protocols, will need to be overcome to strengthen the evidence for clinical application.

### 4.1. Cellular and Molecular Mechanism of Action of PBM

Tissues exposed to PBM absorb the specific wavelength of light through the mitochondrial respiratory chain enzyme, cytochrome C oxidase (COX) [90]. COX, together with porphyrins and heme proteins, plays a crucial role in the absorption of light [91]. According to Karu et al. [92], the action of COX is maximised at wavelengths of 580–700 nm. COX absorbs photon energy (light), which initiates photochemical and photophysical cascades, stimulating the production of ATP [7,85,93]. Mosca et al. [85] revealed that the action of COX generates intracellular ROS and leads to the accumulation of cyclic AMP (cAMP) [93]. The activity of COX is inhibited when nitric oxide (NO) binds to its oxidized or reduced binuclear sites [8]. PBM stimulates the disassociation of NO from COX, therefore reversing the inhibition of NO on COX binding sites, leading to changes in oxidative stress on the mitochondrial membrane [8,18]. These processes activate a cascade of cellular events, leading to physiological changes in cellular signalling, gene transcription, and downstream protein synthesis for cell proliferation, differentiation, and migration [85,94]. Additional proposed effects resulting from the elevation in ATP synthesis leads to an elevation in the activity of Ca^2+^, potassium (K^+^), and sodium (Na^+^) ion transportation, which affects cellular signalling pathways through the action of transcription factors, such as nuclear factor-kappa B (NF-κB) and MAPKs [8,95,96].

### 4.2. Effects of PBM on Cellular Signalling

**Figure 2:**
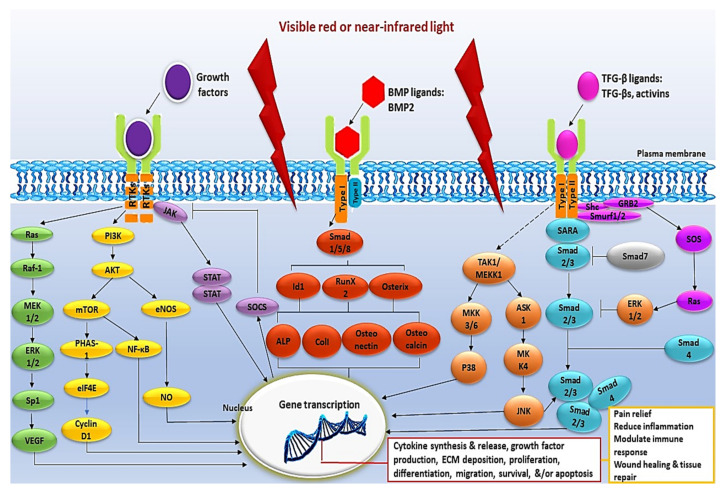
Schematic representation of the intracellular signalling pathways activated by PBM. The intracellular signalling pathways activated by PBM include the MAPK, PI3K/Akt/mTOR, JAK/STAT, BMP/Smad, and TFG-β/Smad pathway. Intracellular signalling pathways are activated in response to a wide array of extracellular signals and are frequently activated in parallel. They are integrated by positive and negative feedback and generate numerous biological signals that depend on the stimulus and on the activated cell type.

Numerous studies have verified the significant role PBM plays in the activation of cellular signalling pathways (Table 2 and Figure 2).

A study conducted by Golovynska et al. [97] reported on the use of PBM with different wavelengths (650, 808, and 1064 nm) on neuronal, human cervical cancer (HeLa), and neuroblastoma cells. The findings suggest that PBM at 650 and 808 nm (3, 6, 12 J/cm^2^) significantly increase cell viability. PBM at 650 and 808 nm (100 mW/cm^2^) increased Ca^2+^ levels regardless of the cell type, thus activating the Ca^2+^ signalling pathway. Huang et al. [98] irradiated mouse primary cortical neurons (810 nm; 25 mW/cm^2^; 3 J/cm^2^) to assess if PBM could protect these neuronal cells against excitotoxicity produced by kainite, glutamate, and N-methyl-D-aspartate (NMDA) in vitro. PBM significantly increased ATP and mitochondrial membrane potential (MMP), and reduced intracellular Ca^2+^, ROS, and NO levels. This highlights the various benefits associated with the use of PBM in regulating cellular signalling pathways. In another study, Sharma et al. [99] reported on the use of PBM in the NIR spectrum (810 nm; 25 mW/cm^2^) at different fluencies (0.03, 0.3, 3, 10, or 30 J/cm^2^). Their findings demonstrated a significant increase in Ca^2+^ levels and MMP, and increased synthesis of ATP, with a biphasic pattern in mice cortical neurons when exposed to lower fluencies. ROS and NO exhibited a double peak pattern, one at low fluencies and another at a high fluence. Lower fluencies were more capable of inducing mediators of cell signalling pathways. Moreover, elevated cellular Ca^2+^ affects numerous signalling pathways, including the MAPK pathway [57].

Shingyochi and colleagues [100] used a carbon dioxide (CO_2_) laser operating at a wavelength of 10.6 μm on human dermal fibroblasts at various fluencies (0.1, 0.5, 1.0, 2.0, or 5.0 J/cm^2^). Their findings suggested that the best treatment option was at a fluence of 1.0 J/cm^2^, which activated the protein kinase B (Akt), ERK, and JNK signalling pathways, thus inducing fibroblast proliferation, migration, and accelerated wound healing [100]. Kawano et al. [101] observed that irradiation of immortalised granulosa (KGN) cells with a gallium-aluminium-arsenide (GaAIAs) laser (830 nm; 60 mW; 60 s irradiation) increases vascular endothelial growth factor (VEGF) production and stimulates MAPK activity. They established that PBM induced VEGF production through the MAPK and ERK signalling pathways. VEGF is a potent pro-neoangiogenic cytokine [109], essential for neovascularization during the wound restorative process [101]. In another study, El-Makakey et al. [102] conducted a comparative study monitoring the physiological effects of pulse electromagnetic field therapy (PEMFT) and PBM on the human body. They reported on the use of a pulsed wave diode laser at a wavelength of 905 nm with an impulse duration of 100 ns and a peak power of 25 W. The 25 patients to whom PBM alone was applied were subjected to the treatment twice daily for six successive days at the site of the appendectomy wound (3–4 cm). Expression levels of MAPK was determined in peripheral lymphocytes. The findings indicated that there was a significant increase in ERK expression levels but not in the expression levels of p38 and JNK [102].

Epidermal growth factor (EGF) plays a critical role in wound healing and is involved in cell proliferation and migration [110]. The interaction between EGF and EGF receptor (EGFR) induces receptor dimerisation and tyrosine autophosphorylation, thereby initiating the JAK/STAT signalling pathway [111]. The JAK/STAT pathway mediates a wide array of cellular responses and is also responsible for the transcription of genes involved in cellular migration, proliferation, and differentiation [76]. Jere and colleagues [21] irradiated human skin fibroblasts at a wavelength of 660 nm (11 mW/cm^2^; 5 J/cm^2^). They reported an increase in the release of EGF, which resulted in the activation of EGFR. This led to activation of the JAK/STAT signalling pathway (increased phosphorylation of JAK2, STAT1, and STAT5), which ultimately led to increased cell proliferation, migration, and viability of diabetic wounded fibroblasts. In a similar study, Jere et al. [110] irradiated wounded and diabetic wounded fibroblast cells (660 nm; 11 mW/cm^2^; 5 J/cm^2^) to profile for 84 JAK/STAT signalling-related genes. They reported that 19 genes were significantly regulated in wounded fibroblast cells (2 genes upregulated and 19 genes downregulated). Diabetic wounded cells responded more favourably, with the upregulation of 46 genes (27 genes downregulated). Of the 46 genes that were upregulated, EGFR, JAK3, STAT1, STAT2, STAT3, STAT5A, and STAT6 were affected. Genes in both models that were downregulated included inhibitors of the JAK/STAT pathway [110]. Both these studies showed that PBM in the visible red spectrum (660 nm) with 5 J/cm^2^ activates cellular signalling by regulating transcription, translation, and activation of proteins involved in the JAK/STAT signalling pathway in wounded and diabetic wounded fibroblast cells [21,110].

TGF-β ligands serve a key role in regulating a variety of physiological processes, such as cellular differentiation, proliferation, growth, and ECM deposition [112]. Additionally, TGF-β isoforms initiate Smad2 and Smad3 phosphorylation, thereby initiating the Smad signalling pathway [113]. The Smad signalling pathway mediates a wide array of biological responses, including stimulating differentiation, proliferation, and apoptosis in various organs [114]. Dang et al. [103] irradiated two-month-old rats with a diode laser (800 nm) at different fluencies of 20, 40, and 60 J/cm^2^, which was performed four times at an interval of two days. The findings suggested that the best treatment to enhance the expression of new collagen was at 40 J/cm^2^. This demonstrated a significant increase in the expression levels of TGFβ and Smad2, 3, and 4. It was also reported that there was an increase in phosphorylated (p)-Smad levels, ultimately activating the TGF-β/Smad signalling pathway and enhanced collagen synthesis [103]. On the contrary, Mokoena et al. [104] irradiated human dermal fibroblasts at a wavelength of 660 nm (12.2 mW/cm^2^; 5 J/cm^2^). The study demonstrated a significant increase in viability, with no stimulation of the TGF-β/Smad pathway. PBM at the reported parameters had no effect on TGF-β1, phosphorylated TGF-β receptor 1 (p-TGF-β1R1), and p-Smad2/3. However, PBM was effective in stimulating the differentiation of fibroblasts into myofibroblasts as was demonstrated by a decrease in the fibroblastic marker Thy-1 (CD90), and an increase over time in extra domain A fibronectin (EDA-FN), a marker of proto-myofibroblasts, and alpha-smooth muscle actin (α-SMA), a marker of myofibroblasts. This process appeared to occur independently of the TGF-β1/Smad signalling pathway [104]. Hirata and colleagues [105] demonstrated that an increase in BMP2-induced alkaline phosphatase (ALP) activity stimulated the phosphorylation of Smad1/5/8, and enhanced the expression of BMP/Smad transcription factors. They concluded that PBM at 805 nm (2.5 W; for 2 min; 5.90 J/cm^2^) can stimulate and regulate the BMP/Smad signalling pathway. According to Shi and Massague [115], phosphorylation of Smad1, 5, and 8 is orchestrated by BMP receptors and propagates their signals, while Smad2 and Smad3 are phosphorylated and activated by TGF-β receptors and mediate their signals.

The phosphatidylinositol 3-kinase (PI3K)/protein kinase B (Akt) pathway is an intracellular signal transduction pathway, which facilitates cell cycle regulation, proliferation, apoptosis, and angiogenesis [116]. PBM has been shown to stimulate the PI3K and Akt signalling pathways. Zhang and colleagues [106] irradiated African green monkey kidney fibroblast cells at 632.8 nm (10 mW; 12.74 mW/cm^2^) at different fluencies (0, 0.2, 0.4, 0.8, and 1.2 J/cm^2^). They demonstrated an increase in cell viability and proliferation, and it was concluded that the best irradiation dose was 1.2 J/cm^2^. In another study, Agas and colleagues [107] irradiated mouse calvaria pre-osteoblasts at 980 nm with fluencies of 45, 27, and 12 J/cm^2^, and power densities of 0.75, 0.45, and 0.20 W/cm^2^, respectively. A fluence of 45 J/cm^2^ and a power density of 0.75 W/cm^2^ demonstrated an increase in pre-osteoblast proliferation and viability, which in turn lead to the activation of the PI3K/Akt/Bcl-2 signalling pathway. A study conducted by Rajendran et al. [108] reported on the use of PBM at 660 (11.2 mW/cm^2^; 5 J/cm^2^) or 830 nm (10.35 mW/cm^2^; 5 J/cm^2^) on diabetic wounded fibroblast cells in vitro. The findings suggested that PBM significantly increased cell viability and reduced oxidative stress. Twelve hours post-PBM with both 660 and 830 nm, there was significantly increased Akt levels and decreased FOXO1 levels. There was also an increase in the enzymic antioxidants catalase (CAT), superoxide dismutase (SOD), and heme oxygenase (HMOX1) 24 h post-irradiation at both wavelengths. FOXO1 is downstream of Akt and is inhibited when Akt is activated, leading to increased cell survival and decreased apoptosis. Antioxidants have a substantial role in maintaining a normal redox balance by removing free radicals and ROS. Under hyperglycaemic conditions, there is increased generation of free radicals and ROS, resulting in an unbalanced redox state. This study showed that PBM at 660 and 830 nm with 5 J/cm^2^ was able to rectify a hyperglycaemic-induced redox imbalance and attenuate oxidative stress possibly through activation of the Akt/FOXO1 signalling pathway [108].

## 5. Conclusions

Wound healing is a multifaceted multistage process coordinated by various cell types across multiple tissues and organs to address an acute need. Many factors can interfere with the proper healing of wounds, leading to the development of chronic wounds. These non-healing chronic wounds pose a major global challenge to healthcare systems and impose undue stress on patients and their families and caregivers. Various positive responses to PBM in wound healing have been noted over the years in vitro and in vivo. PBM leads to photochemical reactions and the production of ATP activates a cascade of cellular events, which in turn leads to biological changes and downstream effects. These effects include, but are not limited to, the restoration of cellular function, mitigation of inflammation and pain, augmentation of tissue regeneration and wound repair, reduced oxidative stress, immunomodulation, neuronal regulation, accelerated cell proliferation and migration, stimulated release of growth factors, and promotion of ECM synthesis [8,117]. The upregulation of intracellular signalling pathways is required in impaired wound healing where normal functionality is compromised. Such pathways are activated in response to a wide array of extracellular signals. As a result, extensive research on the impact of PBM on cellular responses involved in wound healing is constantly conducted to obtain scientific, reliable results. PBM has been found to have an influence over and stimulate the ERK1/2 (MAPK), Wnt, JAK/STAT, PI3K/AKT, TGF-β/Smad, Notch, JNK/MAPK, and P38/MAPK pathways in various cell types and under different conditions. Further research into specific signal transduction pathways and activation of defined molecules in these pathways is an important step in understanding how PBM affects cellular processes, such as differentiation, migration, and proliferation. Such studies will further promote and advance the field of PBM. Therefore, obstacles, such as the shortage of translational research with consistent protocols, will need to be overcome to strengthen and advance the reliability and validity for clinical application.

## Figures and Tables

**Table 1:** An overview of signalling pathways involved in acute and chronic wounds and their response to PBM.

Pathway	Role	Cell Type	Acute Wounds	Phase of Healing	Chronic Wounds	Response to PBM	References
ERK1/2 MAPK	Cell growth, cell cycle, proliferation, and differentiation	Endothelial, keratinocyte, fibroblast cells	Up- regulated	Proliferation, remodelling	Down -regulated	Up- regulated	[15,16]
Wnt	Proliferation, migration, and cell self-renewal	Epithelial, stem, fibroblast cells	Up- regulated	Every subsequent phase	Down -regulated	Up- regulated	[17,18,19,20]
JAK/STAT	Cell proliferation and migration	Fibroblast cells	Up- regulated	Inflammation, proliferation	Down -regulated	Up- regulated	[18,21,22]
PI3K/AKT	Cell proliferation and survival	Fibroblast, epithelial, stem cells	Up- regulated	Every subsequent phase	Down -regulated	Up- regulated	[18,23,24]
TGF-β	Cell proliferation, angiogenesis, and wound contraction	Fibroblast, epithelial stem, endothelial cells	Up- regulated	Every subsequent phase	Down -regulated	Up- regulated	[17,18,25]
Notch	Cell proliferation and angiogenesis	Endothelial, keratinocyte, fibroblast, stem cells	Up- regulated	Every subsequent phase	Down -regulated	Up- regulated	[26,27,28]
JNK MAPK	Cell apoptosis, proliferation, and migration	Epithelial, keratinocyte cells	Up- regulated	Every subsequent phase	Down -regulated	Up- regulated	[18,29,30]
P38 MAPK	Cell apoptosis, proliferation, differentiation, cytokine production, neuropathic pain, and survival	Endothelial, keratinocyte, fibroblast cells	Up- regulated	Inflammatory, proliferation	Down -regulated	Up- regulated	[18,31,32,33]

**Table 2:** The effects of PBM on cellular signalling.

Wavelength (nm)	Dose (J/cm^2^)	Power Density (mW/cm^2^)	Studied Model	Outcomes/Observations	Reference
650, 808 and 1064	3, 6 and 12	100	Neurons, HeLa, and neuroblastoma	Significant increase in cell viability, Ca^2+^ levels and ROS generation at a wavelength of 650 and 808 nm.	[97]
810	3	25	Mouse primary cortical neurons	Increased cellular viability, ATP, and MMP in all three excitotoxins. Decreased intracellular Ca^2+^, ROS, and NO in all three excitotoxins.	[98]
810	0.03, 0.3, 3, 10 and 30	25	Mice primary cortical neurons	Significant increase in Ca^2+^, MMP and ATP at a fluence ≤ 3 J/cm^2^. 10 and 30 J/cm^2^ decreased Ca^2+^, MMP and ATP. Significant increase in ROS at a fluence of ≤3 J/cm^2^. At a fluence of 30 J/cm^2^, there was a second increase in ROS and NO induction.	[99]
10600	0.1, 0.5, 1.0, 2.0 or 5.0	52.08 520.83	Human dermal fibroblasts	A fluence of 1.0 J/cm^2^ increased cell proliferation, migration, and activation of Akt, ERK, and JNK.	[100]
830	-	-	Immortalised granulosa (KGN) cells	Significant increase in the release of VEGF, and activation of p44 and p42 ERKs.	[101]
905	-	-	Appendectomy wound (3–4 cm) on human bodies	Significant increase in ERK expression levels. No increase in P38 and JNK expression levels.	[102]
660	5	11	Human skin fibroblasts	Significant increase in cell proliferation, viability, rate of migration, release of EGF, activation of EGFR, STAT1, STAT5, and JAK2.	[21]
800	20, 40 and 60	-	Female Sprague- Dawley rats	Significant increase in the expression and activation of procollagen type I and IV, TGF-β, Smad2, 3, and 4 at 40 J/cm^2^.	[103]
660	5	12.2	Human skin fibroblast cells	Significant increase in cell viability. No stimulation in p-Smad2/3, p-TGF-β1R1, and TGF-β1. Enhance fibroblast differentiation into myofibroblasts.	[104]
805	2.0–12.0	-	Mouse calvariae myoblast cells	Significant increase in the activation of ALP, Smad1, 5, and 8, and expression of BMP/Smad target genes (Id1, Osterix, and Runx2).	[105]
632.8	0, 0.2, 0.4, 0.8 and 1.2	12.74	African green monkey kidney fibroblast cells	Significant increase in cell viability, proliferation, and activation of Akt at ≥0.4 J/cm^2^.	[106]
980	12, 27 and 45	200, 450 and 750	Mouse calvaria pre-osteoblasts	A fluence at 45 J/cm^2^ increased cell proliferation, viability, and regulation of the PI3K/Akt/Bcl-2 signalling pathway.	[107]
660 830	5	11.2 10.3	Human skin fibroblast cells	Significant increase in cell viability. Increased activation of Akt and antioxidant levels (SOD, CAT, HMOX1), and decreased FOXO1 levels.	[108]
